# Explainable deep learning-based comparative study for guava fruit and leaf disease classification: advancing agricultural diagnostics through AI

**DOI:** 10.3389/fpls.2026.1738585

**Published:** 2026-04-28

**Authors:** Zeyu Zhou

**Affiliations:** School of Economics, Shandong Normal University, Jinan, Shandong, China

**Keywords:** deep learning, explainable AI, Grad-CAM, guava disease classification, hybrid CNN, plant pathology, smart agriculture, transfer learning

## Abstract

**Introduction:**

Early detection of plant diseases is essential for maintaining crop health and ensuring sustainable agricultural productivity. Guava fruit and leaf diseases, if not identified at an early stage, can lead to significant yield losses. Recent advances in deep learning offer promising solutions; however, challenges remain in achieving both high accuracy and model interpretability for practical agricultural deployment.

**Methods:**

This study proposes an explainable deep learning–based framework for the classification of guava fruit and leaf diseases. A real-world dataset consisting of 527 annotated images across five classes—Disease Free, Phytophthora, Red Rust, Scab, and Styler and Root Rot—was utilized. Six hybrid model architectures were developed by integrating transfer learning backbones (VGG16, MobileNetV2, InceptionV3, and ResNet50) with custom convolutional neural network (CNN) classifiers. Model performance was evaluated using accuracy, precision, recall, F1-score, and class-wise metrics. To enhance transparency, Gradient-weighted Class Activation Mapping (Grad-CAM) was employed to visualize disease-relevant regions.

**Results:**

Among all evaluated models, the proposed VGG16 + MobileNetV2 hybrid architecture achieved the best performance, attaining an accuracy of 96%, an F1-score of 0.96, and strong generalization across all disease classes. Comparative analyses using confusion matrices, ROC-AUC curves, precision–recall curves, and radar plots confirmed the superior and consistent performance of the proposed model over other hybrid configurations.

**Discussion:**

The results demonstrate that combining deep feature extractors with lightweight architectures enhances both classification accuracy and computational efficiency. The integration of Grad-CAM provides meaningful visual explanations, increasing trust and interpretability in AI-assisted disease diagnosis. This framework shows strong potential for deployment in real-time smart farming systems and mobile-based diagnostic applications, particularly in resource-constrained agricultural environments.

## Introduction

Plant diseases represent a substantial threat to global food security and the economic stability of farmers, particularly smallholder farmers in developing nations, where agricultural production is heavily reliant on healthy crops (Mohanty et al., 2016). Accurate and timely disease identification is crucial for implementing effective disease management strategies, minimizing crop losses, and reducing the need for extensive pesticide applications (Mohanty et al., 2016). Traditional methods of plant disease diagnosis, which often depend on visual inspection by agricultural experts or laboratory analysis, are time-consuming, expensive, and prone to subjective errors (Mohanty et al., 2016).

To counter these shortcomings, the growing popularity in using advanced technologies to achieve automation and enhanced accuracy of plant disease detection has played the role of deep learning (Shoaib et al., 2025). Deployed deep learning approaches showed extraordinary success in a variety of computer vision duties, such as picture classification, object detection, and semantic segmentation (Chen et al., 2025). The techniques provide the promise of examining the complex patterns in plant images that could be then used to identify delicate disease symptoms that would be otherwise overlooked by human observer.

Guava plants are especially susceptible to various fungal, bacterial, and viral infections, which may also have severe effects on the yield of the fruit and their quality. These diseases require early and accurate detection so that some timely action could be implemented to prevent any wide range of outbreaks. The contemporary technologies allowed human societies to generate sufficient food supplies to cover the needs of the population of more than 7 billion people; nevertheless, several problems, such as plant diseases, still corrode food security (Mohanty et al., 2016). Identification of the disease at the first level is one of the critical conditions in the process of effective disease management, with or without the method applied (Mohanty et al., 2016).

The problem of guava disease is a massive threat to food security at a global level (Amin et al., 2022). Conventional methods in plant disease detection are based on professional experience which is both time consuming and costly. The approaches can also be unrealistic in small farms and developing nations (Fedoroff, 2015). The new creation of the image processing methods has resulted in increased research on the identification of plant diseases (Ngugi et al., 2020).

The application of deep learning models presents a strong response, although there are a few obstacles to their use in an agricultural context, e.g. computational requirements, interpretability. Deep learning models are usually very complex and thus they can take up a lot of computational power and cannot be implemented in a resource-limited device, or even in a place where internet connectivity is not wide-spread.

The ability of deep learning to perform feature learning is of particular interest, since systems can now learn complex disease properties on image data without any need to engineer features by hand (Saleem et al., 2020). The effectiveness of the deep learning networks highly depends on the training data size and quality, and training using large datasets of diverse samples is needed to avoid overfitting and introduce versatility among various conditions of the environment in the model decisions (Sinamenye et al., 2025). Deep learning models make it possible to easily classify diseases even on the devices like smartphones (Mohanty et al., 2016). This has the potential to facilitate real-time diagnosis of a disease at the field level that would enable an immediate intervention and containment efforts (Mohanty et al., 2016).

Nevertheless, deep learning models tend to work like a black box, so it is hard to explain why it refers to specific reasoning when making predictions. Such lack of transparency leaves doubts on trust and acceptance especially where the application can cause very important decisions. They are exploring explainable AI tools to solve this problem because those tools can demonstrate how decisions have been made by deep learning models (Dolatabadian et al., 2024). Explainable AI has the potential to improve the trust in the predictions and help model debugging, as well as provides explanatory information regarding the underlying biology of plant diseases by highlighting their characteristics and trends. Application of explainable AI in enhancing the generation and establishment of deep learning models in agriculture. This study aims at exploring the applicability of explainable deep learning strategies in categorizing the diseases pastured on guava fruits and leaves. The research aims to compare and analyze the use of different deep learning models, such as convolutional neural networks, to recognize and classify widespread diseases in guava basing on the images of leaves and fruits (Wang et al., 2017). The study will also be focused on the creation and introduction of explainable AI to provide explainability into why the deep learning models arrived at their final outcome, increasing the reliability and accountability of their diagnoses.

The logic behind how the deep learning models work is not clear when they are applied in most contexts, and thus their level of acceptance may be hampered in the cases of application in areas such as agriculture where reasons of a decision ought to be justified and comprehended (Singh et al., 2020). Developing a way to visualize, interpret, explain, and understand deep learning models has attracted much recent attention, since their nested non-linear latent space has tended to be perceptual as a black box that provides no insight into the variables affecting predictions made by the model and most actions follow this definition of perceptual. Unlike in case with traditional AI models, the farmers have little confidence in the recommendations generated by AI systems and the ability to make an informed decision based on the recommendations (Chen et al., 2023).

Explainable AI methods are essential for addressing these issues because they make AI systems more transparent, understandable, and trustworthy (Muhammad and Bendechache, 2024; Samek et al., 2017; Samek and Müller, 2019; Zacharias et al., 2022). Trust and Reliability: Providing explanations for deep learning model predictions is crucial for building trust and ensuring the reliability of AI-driven disease diagnostics in agriculture. By revealing the underlying factors that contribute to a disease diagnosis, explainable AI enables farmers and agricultural experts to validate the model’s predictions and gain confidence in its accuracy. Explainable AI can help to ensure fairness and prevent unintended biases in AI systems.

The research will continue with the agenda on agricultural diagnostics to create an understandable and implementable deep-learning system to classify guava diseases. The framework will synergize state-of-art deep-learning architecture and explainable AI approaches, offer high-fidelity diagnosis of the disease and the actionable intelligence on the factors underlying the diagnosis. One can find and correct the biases in the AI systems through explainable AI techniques, and can also correct bias in the predictions of the system that helps in predicting fair and equitable anticipation among demographic segments or geographic areas. The interpretability of the model matters because it can be cumbersome to decipher the logic behind a certain prediction by a machine learning algorithm, and this may become a source of irregularity and uncertainty in decision-making (Edunjobi and Odejide, 2024). The interpretation of AI models ExAI can be used to further clinical decision support systems (Rane et al., 2023). Due to the high access of structured and unstructured data and rapid growth in analytics methods, the healthcare sector is on the verge of revolution (Dave et al., 2020). Explainable AI provides insight to the stakeholders with regard to the methodologies used by the AI algorithms to make decisions, predictions and implement their operations (Chang et al., 2024). This allows understanding what drives the primary decisions in the models (Chang et al., 2024). This study will aim at the following.

1. To develop a reliable deep learning-based system for classifying guava leaf and fruit diseases using visual image data collected from a curated and well-annotated dataset.
2. To conduct a comparative analysis of multiple convolutional neural network (CNN) architectures, including VGG16, ResNet50, InceptionV3, MobileNetV2, and custom hybrid models, to evaluate and benchmark their classification performance on guava disease identification.
3. To enhance model transparency and interpretability by incorporating Explainable Artificial Intelligence (XAI) techniques, specifically Gradient-weighted Class Activation Mapping (Grad-CAM), to visualize and understand the decision-making process of the trained models.
4. To assess model effectiveness using comprehensive performance metrics such as accuracy, precision, recall, F1-score, and visualization of confusion matrices to determine the most suitable architecture for practical agricultural deployment.
5. To contribute a reproducible and interpretable AI pipeline that supports early disease diagnosis in guava cultivation, thereby promoting precision agriculture and improving crop health management through explainable deep learning models.

## Methodology

This section elaborates the comprehensive methodology employed in our research to detect and classify guava fruit and leaf diseases using deep learning-based hybrid models with explainability. The approach encompasses dataset preparation, preprocessing, hybrid model construction, training strategy, and interpretability.

### Dataset description

The dataset (Abdulazeem, 2023) used in this study was collected and organized into two subsets: Train and Test, comprising a total of 527 RGB images. The data represents real-world guava crop conditions and includes both healthy and diseased samples of fruits and leaves. The images are categorized into the following five distinct classes, each corresponding to a unique plant condition:

1. Disease Free (Healthy)
2. Phytopthora (a severe fungal infection affecting leaves and roots)
3. Red Rust (a common fungal infection presenting reddish-brown spots)
4. Scab (a disease that causes scabby lesions on fruit and leaf surfaces)
5. Styler and Root Rot (a condition involving multiple root-related fungal infections)

**Figure 1:**
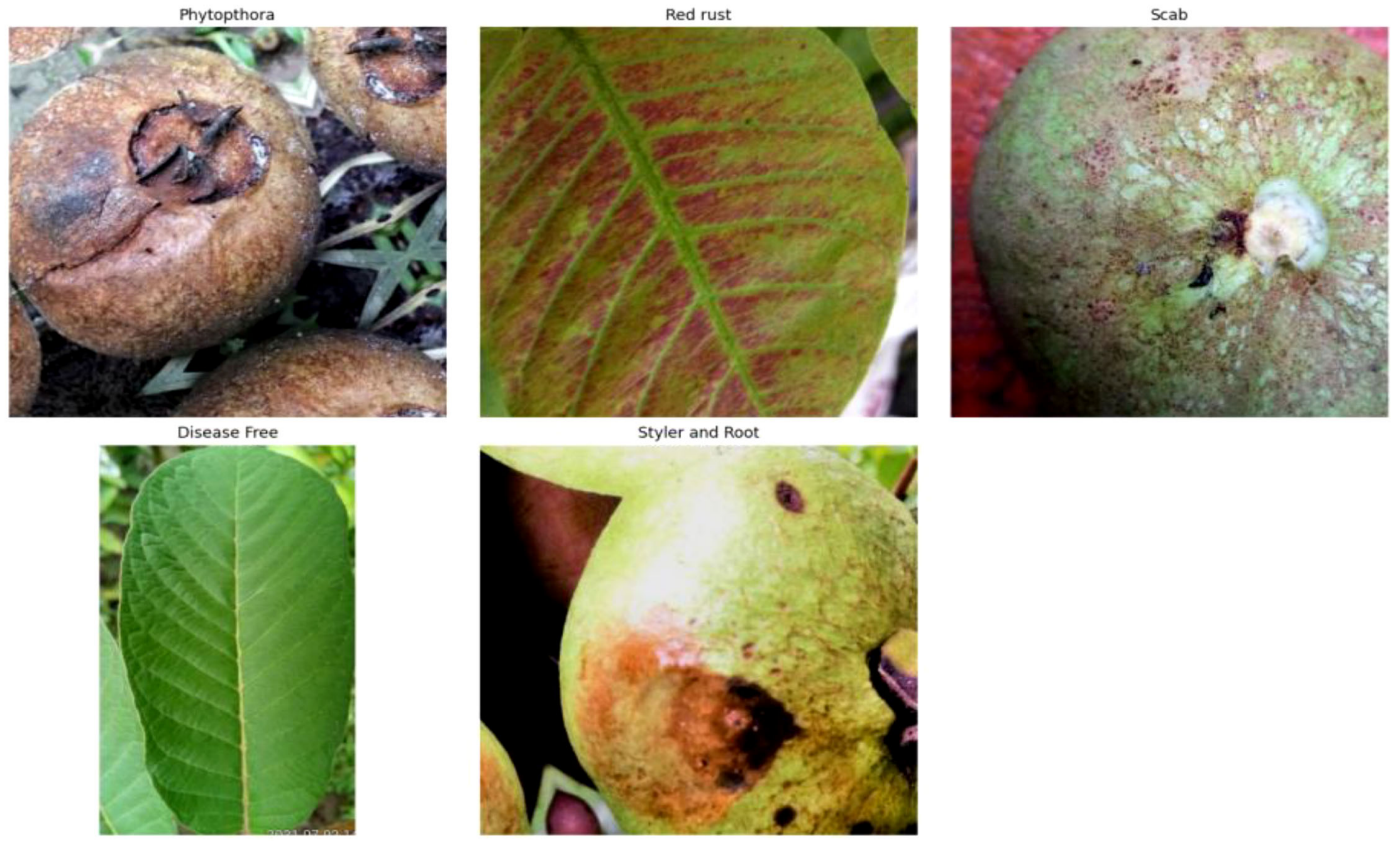
Sample images from the five guava disease classes in the dataset.

The **Figure 1** shows representative RGB images for each disease category, highlighting variations in texture, color, and symptom presentation across guava leaves and fruits. These examples reflect the real-world complexity of plant disease identification.

The dataset was curated to maintain class diversity and minimize bias. **Table 1** provides the breakdown of image distribution:

**Table 1:** Class-wise distribution of train and test images.

Class name	Training samples	Testing samples
Disease Free	102	26
Phytopthora	96	23
Red Rust	83	18
Scab	101	22
Styler and Root	37	19
Total	419	108

The dataset was collected under variable lighting conditions and backgrounds to simulate realistic field data. This introduces natural noise and complexity, ensuring that the model learns robust features. The image resolution varied originally, but all images were uniformly resized during preprocessing.

### Data preprocessing

To prepare the dataset for model ingestion, a rigorous preprocessing pipeline was applied:

#### Resizing

All images were resized to a uniform dimension of pixels:


Iresized=Resize(Ioriginal,(224,224))

#### Normalization

Pixel values were normalized to:


$${\text{I}}_{\mathrm{norm}}=\frac{{\text{I}}_{\mathrm{resized}}}{255}$$

#### One-hot encoding

Labels were encoded into one-hot vectors:


y=OneHotEncode(C), C∈{0,1,2,3,4}

#### Data augmentation

The training images were augmented using:

- Horizontal & vertical flips
- Rotations (± 30°)
- Zoom (0.8–1.2)
- Width/height shifts (± 10%)

#### Stratified shuffling

Ensured balanced representation per batch.

### Proposed model architecture

Six hybrid models were developed combining transfer learning backbones with custom fully connected layers. The final proposed model was a combination of VGG16 + MobileNetV2, integrating both low-level texture features and high-level spatial features.

The architecture consists of the following layers:

#### Step 1: dual input branches

We input the same resized RGB image $x\;\in \;{\mathbb{R}}^{224\times 224\times 3}$ to both models.


$$x_{\mathrm{vgg}}=x_{\mathrm{mobilenet}}=x$$

#### Step 2: feature extraction via VGG16 and MobileNetV2

Let ${\text{f}}_{\mathrm{vgg}}(x)$ and ${\text{f}}_{\mathrm{mobilenet}}(x)$ represent the feature maps extracted by each backbone. Both models are pretrained on ImageNet and frozen during early training:


$$\begin{array}{l} F_{\mathrm{vgg}}=f_{\mathrm{vgg}}(x_{\mathrm{vgg}})\in {\mathbb{R}}^{7\times 7\times 512}\\ F_{\mathrm{mobilenet}}=f_{\mathrm{mobilenet}}(x_{\mathrm{mobilenet}})\in {\mathbb{R}}^{7\times 7\times 1280} \end{array}$$

#### Step 3: global average pooling

Both feature maps are downsampled using Global Average Pooling to reduce spatial dimensions:


ɡvgg=GAP(Fvgg)∈ℝ512 ɡmobilenet=GAP(Fmobilenet)∈ℝ1280

#### Step 4: feature concatenation

The pooled feature vectors are concatenated to form a single high-level descriptor:


$${ɡ}_{\mathrm{concat}}=\mathrm{concat}({ɡ}_{\mathrm{vgg}},{ɡ}_{\mathrm{mobilenet}})\in {\mathbb{R}}^{1792}$$

#### Step 5: fully connected dense layer

The combined feature vector passes through a dense layer with 512 units and ReLU activation:


h=ReLU(W1·ɡconcat+b1), W1∈ℝ512×1792

#### Step 6: dropout regularization

A dropout layer is applied to prevent overfitting:


$$h_{drop}=\mathrm{Dropout}(h,p=0.5)$$

#### Step 7: output layer with softmax activation

The final classification layer has 5 neurons (for 5 classes), activated via softmax:


$$\hat{y}=\mathrm{Soft}\max(W_{2}\cdot h_{\mathrm{drop}}+b_{2}),\;W_{2}\in {\mathbb{R}}^{5\times 512}$$

Final Model Summary Equation.


y^=Softmax(W2·Dropout(ReLU(W1·Concat(GAP(fvgg(x)), GAP(fmobilenet(x)))+b1), 0.5)+b2)

**Figure 2:**
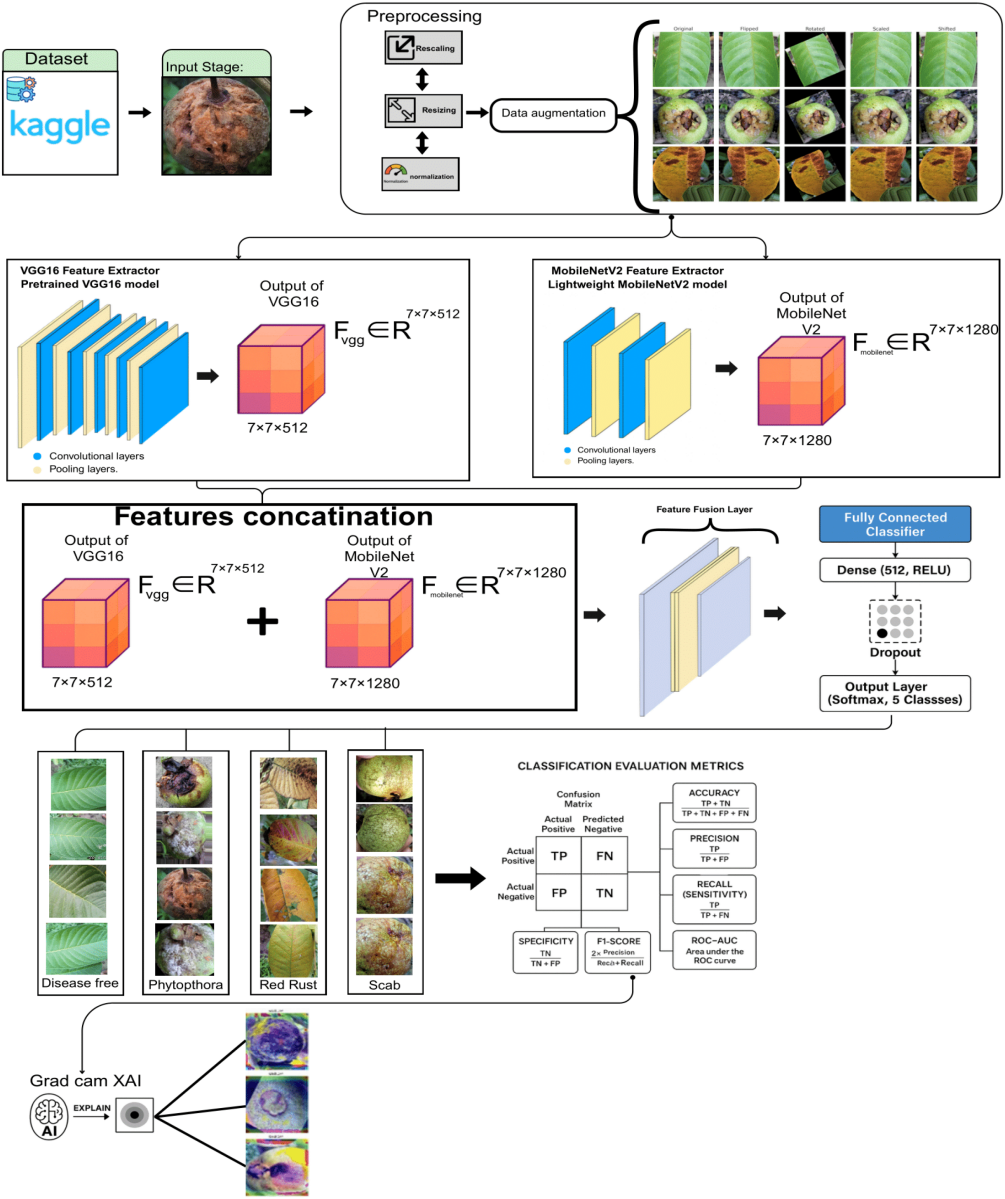
Architectural workflow of the proposed VGG16 + MobileNetV2 hybrid model for guava disease classification.

**Figure 2** presents a comprehensive visual workflow of the proposed hybrid deep learning framework. The pipeline begins with the preprocessing stage, where RGB images of guava fruit and leaf samples are resized, rescaled, and normalized. Augmented versions are generated using flipping, rotation, and zoom to increase training robustness. The input is then simultaneously passed through two parallel branches: a VGG16 feature extractor and a MobileNetV2 extractor. These feature maps are concatenated in the fusion layer, capturing both fine-grained and spatial features. The fused vector is passed through a dense classifier, and Grad-CAM is applied *post hoc* for explainability, highlighting class-relevant regions. This end-to-end workflow enables accurate and interpretable classification into one of the five disease categories: Disease Free, Phytophthora, Red Rust, Scab, and Styler and Root Rot.

**Table 2** summarizes the six hybrid (Fine-tuned) deep learning model combinations evaluated in this study, each integrating two different backbones. The proposed model leverages VGG16 and MobileNetV2 to enhance feature diversity and classification accuracy.

**Table 2:** Hybrid model combinations evaluated.

Hybrid model (Fine-tuned)	Backbone 1	Backbone 2
Model 1 (VGG16+CNN)	VGG16	Custom CNN
Model 2 (MobileNet+CNN)	MobileNetV2	Custom CNN
Model 3 (Inception+CNN)	InceptionV3	Custom CNN
Model 4 (VGG16+ResNet50)	VGG16	ResNet50
Model 5 (Inception+ResNet50)	InceptionV3	ResNet50
Model 6 (Proposed)	VGG16	MobileNetV2

### Evaluation metrics

To assess the model’s performance comprehensively, multiple evaluation metrics were applied:

#### Accuracy

Accuracy measures the overall proportion of correctly classified instances among the total number of samples. It is defined as:

#### Precision

Precision quantifies the proportion of positive identifications that are actually correct. It is particularly useful when the cost of false positives is high.


$$\left[\mathrm{Precision}=\frac{TP}{TP+FP}\right]$$

#### Recall (sensitivity)

Recall indicates the proportion of actual positive cases that are correctly identified by the model. It is crucial when minimizing false negatives is important.


$$\left[\mathrm{Recall}=\frac{TP}{TP+FN}\right]$$

#### F1 score

The F1 score is the harmonic mean of precision and recall. It provides a single metric that balances both concerns, especially useful when class distribution is imbalanced.


$$\left[\mathrm{F1}\;\mathrm{Score}\;=2\times \frac{\mathrm{Precision}\times \mathrm{Recall}}{\mathrm{Precision}+\mathrm{Recall}}\right]$$

Confusion matrix


$$CM_{ij}=Number\;of\;samples\;from\;class\;i\;predicted\;as\;class\;j$$

### Model training

The models were implemented in TensorFlow with GPU acceleration on Google Colab. Key parameters:

Batch Size: 32
Epochs: 50
Optimizer: Adam,
Loss Function: Categorical Crossentropy

$$L_{CE}=-\sum_{i=1}^{5}y_{i}\cdot \log({\hat{y}}_{i})$$

Callbacks: EarlyStopping, ModelCheckpoint
Pretraining: All backbones were loaded with ImageNet weights, initially frozen. Fine-tuning was applied to the top layers.

### Model explainability with Grad-CAM

Explainability was introduced using Grad-CAM to visualize which regions influenced the classification decision. The heatmap is computed as:

## Results

To evaluate the effectiveness of the proposed hybrid deep learning framework for guava disease classification, a comprehensive set of experiments was conducted on a curated dataset comprising five distinct classes of guava fruit and leaf conditions. Six fine-tuned hybrid models were developed by combining state-of-the-art convolutional neural network backbones, including VGG16, MobileNetV2, InceptionV3, ResNet50, and custom CNN layers. Each model was trained under identical conditions and evaluated on the unseen test set of 108 images to ensure fair comparison and reproducibility.

Common measures of classification including, accuracy, precision, recall, and F1-score were used to analyze the results. Besides numerical assessment, visual explanations were incorporated using Grad-CAM in supporting the model attention on disease of interest regions. The suggested model as a combination of VGG16 and MobileNetV2 was always the best among hybrid models proving higher diagnostic accuracy, resistance to class imbalance, and interpretability of the decision-making process. In the following subsections, performance of the various hybrid models is cited and compared.

**Figure 3:**
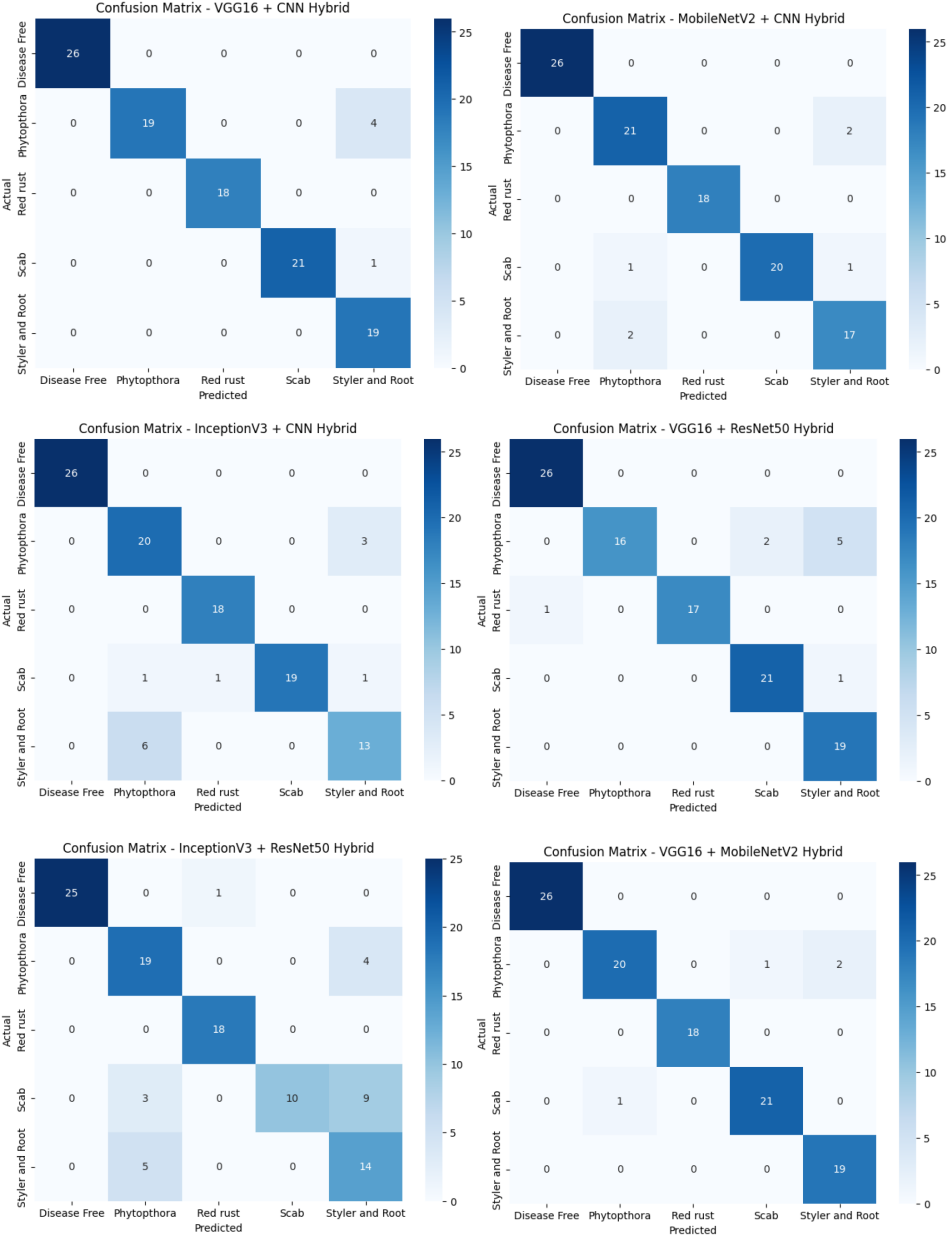
Confusion matrices for all hybrid models evaluated in the study.

The confusion matrices provide a granular view of how each hybrid model performed across the five guava disease classes. True positives are heavily concentrated along the diagonals, indicating strong classification capabilities with minimal misclassification. As shown in **Figure 3**, most models demonstrate high precision for classes like *Disease Free* and *Red Rust*, while certain misclassifications are observed in challenging categories such as *Phytophthora* and *Styler and Root Rot*.

**Figure 4:**
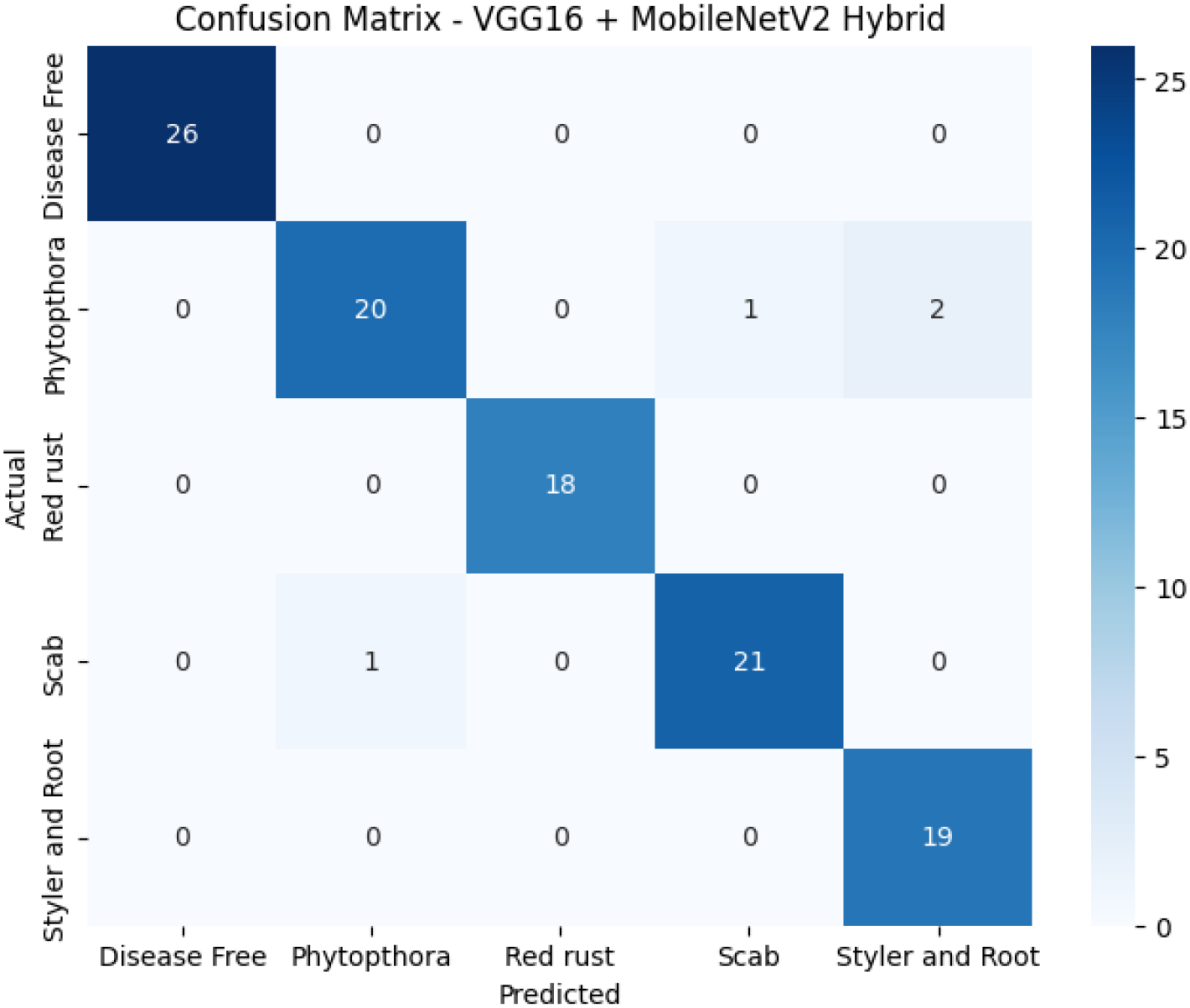
Confusion matrix for the proposed VGG16 + MobileNetV2 hybrid model.

The proposed model exhibits excellent classification performance, with nearly perfect prediction across all classes. As depicted in **Figure 4**, the model achieves perfect precision for *Disease Free*, *Red Rust*, and *Styler and Root*, with minor misclassifications observed only in the *Phytophthora* class.

**Figure 5:**
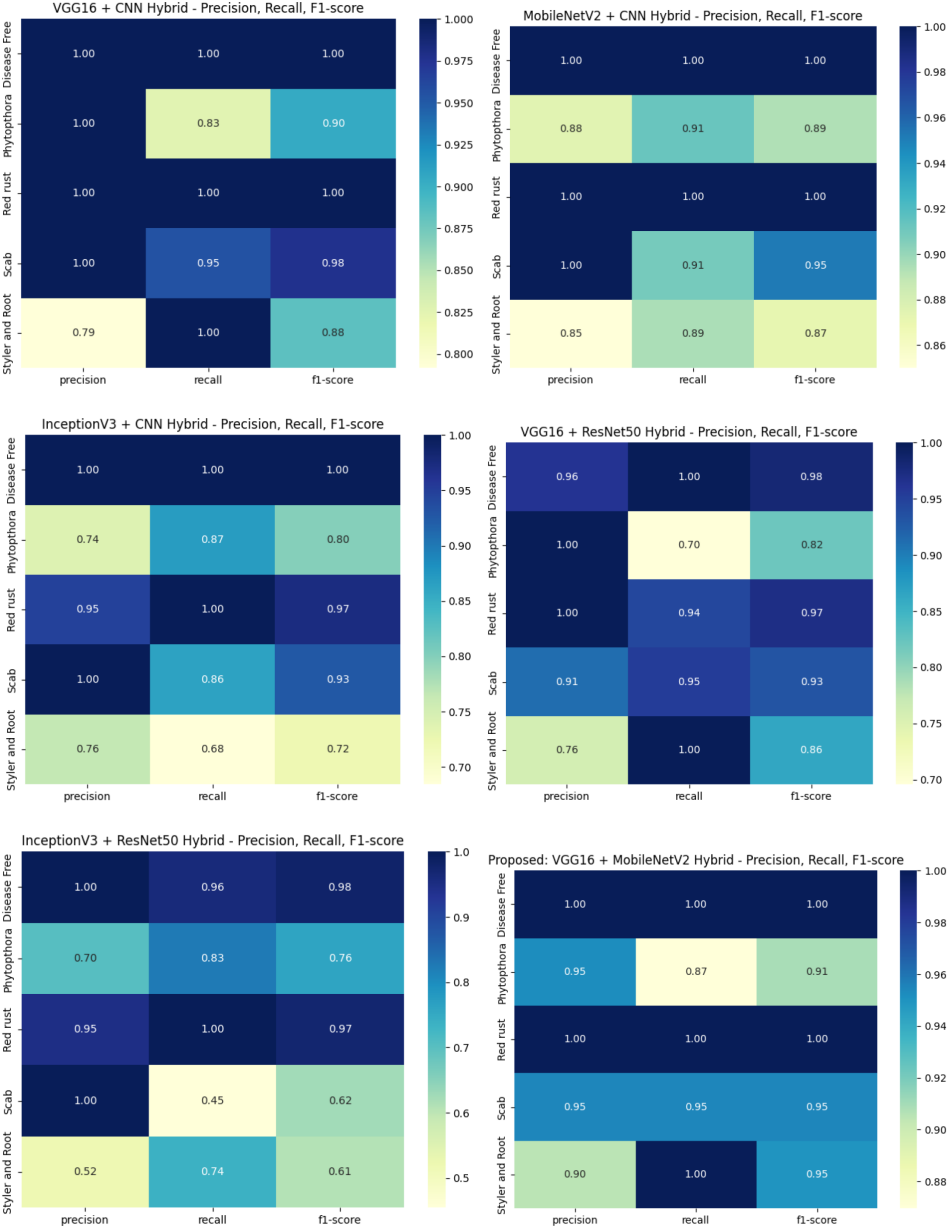
Heatmaps of precision, recall, and F1-score for each disease class across all hybrid models.

As shown in **Figure 5**, the heatmaps provide a comparative overview of each hybrid model’s performance per class. The proposed VGG16 + MobileNetV2 model consistently demonstrates superior scores, especially in the *Red Rust* and *Styler and Root* categories.

**Figure 6:**
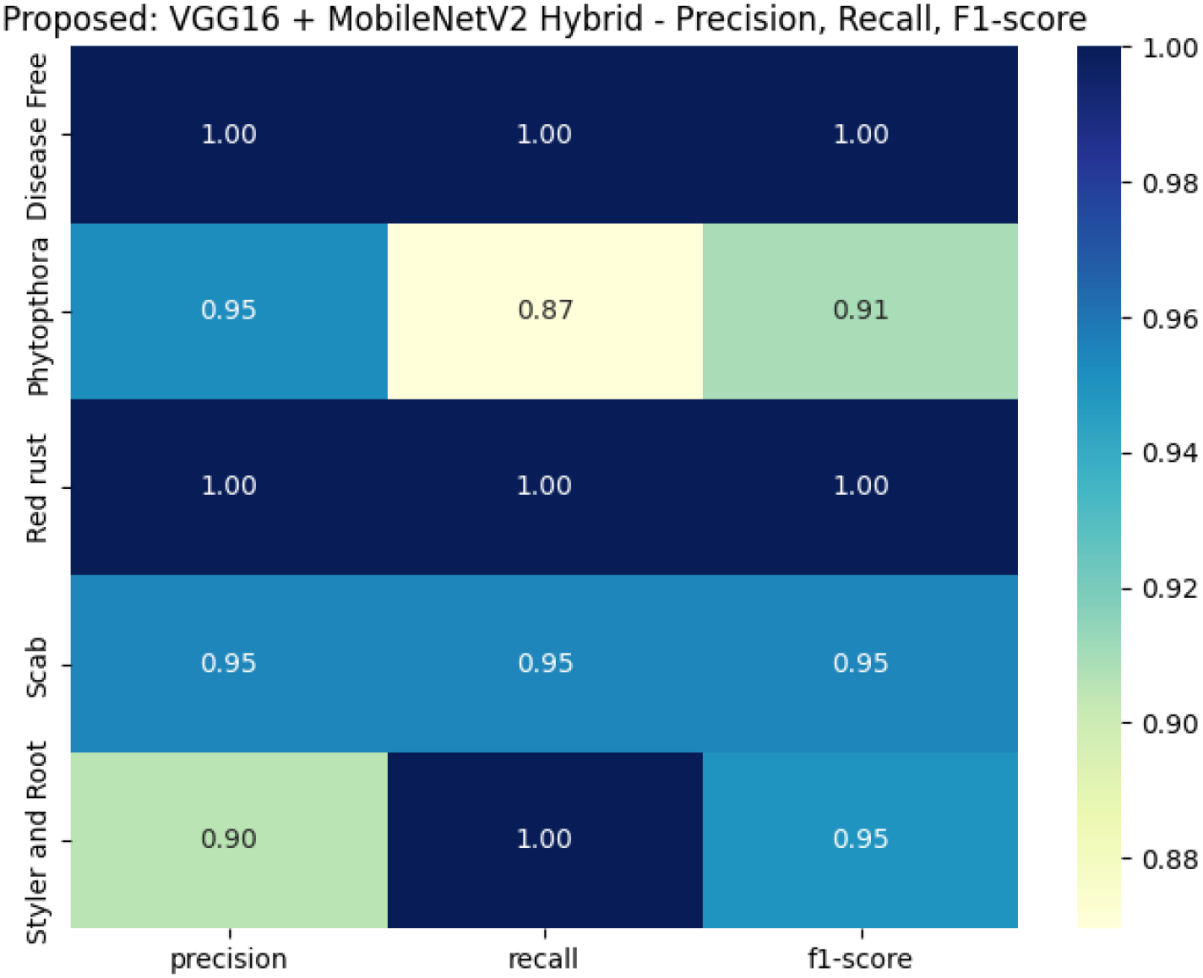
Heatmap of precision, recall, and F1-score for each disease class across all proposed model.

As illustrated in **Figure 6**, the proposed model exhibits highly consistent performance across all metrics, achieving perfect or near-perfect scores in *Disease Free*, *Red Rust*, and *Styler and Root* categories, reflecting its robustness and generalizability.

**Figure 7:**
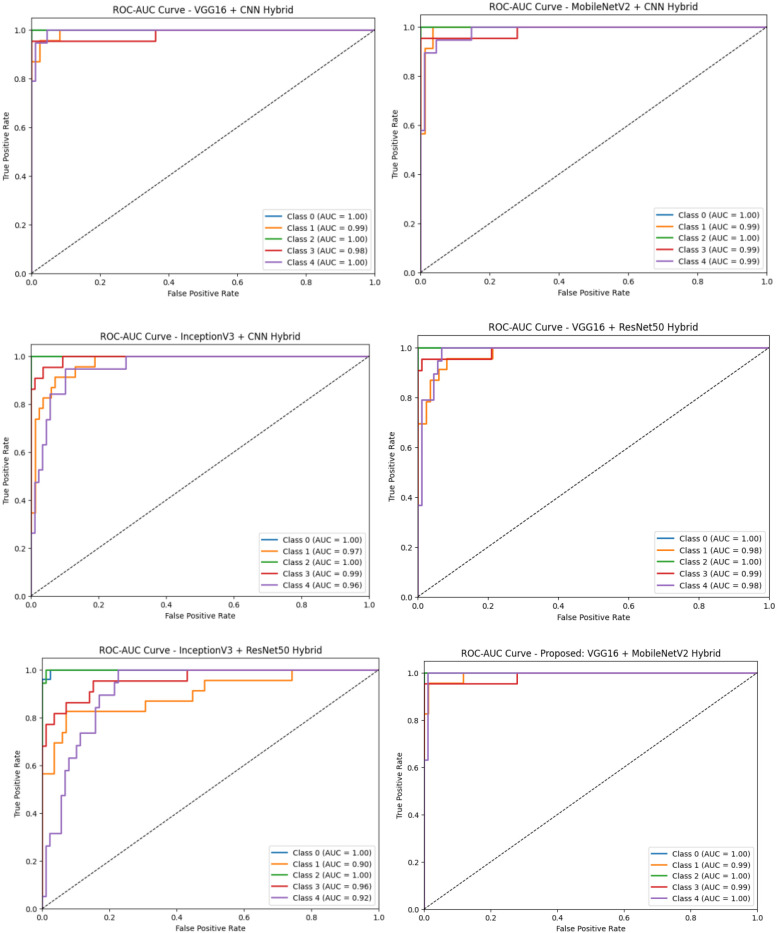
ROC-AUC curves for all hybrid models evaluated, comparing their multi-class classification performance across five guava disease categories.

**Figure 8:**
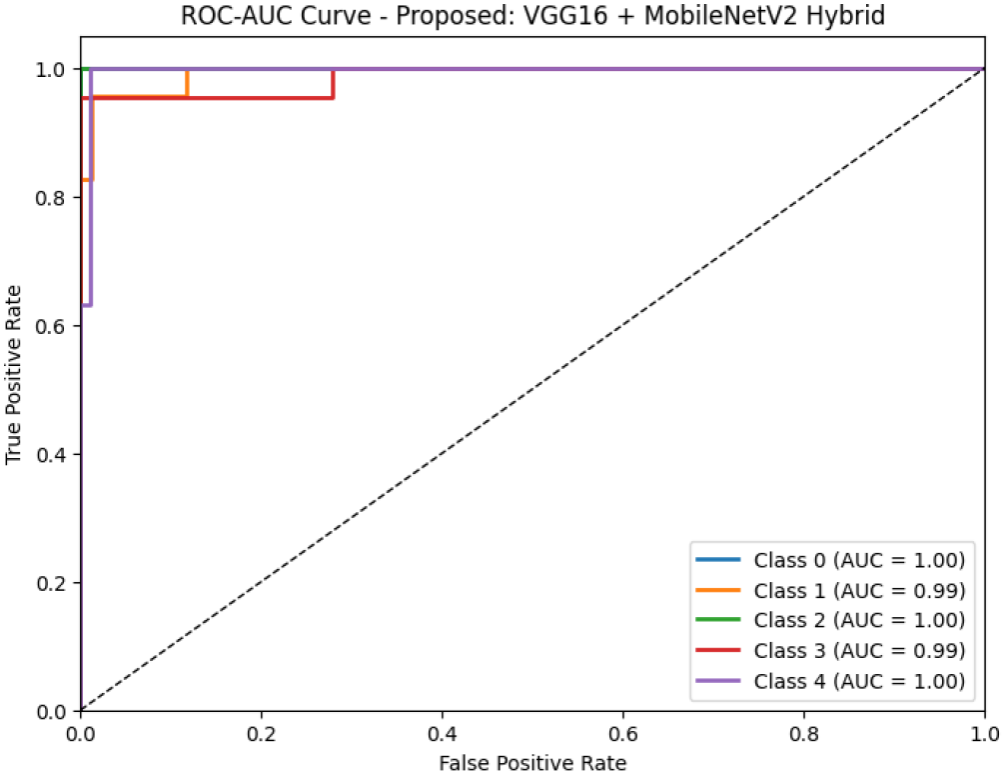
ROC-AUC curve for the proposed VGG16 + MobileNetV2 hybrid model, demonstrating high discriminatory power with near-perfect AUC values for all classes.

**Figure 7** presents the ROC-AUC curves for all hybrid models evaluated in the study, enabling a comparative view of their classification performance across all five disease categories. Models such as VGG16+CNN and MobileNetV2+CNN demonstrate strong discriminative capabilities, while the proposed VGG16+MobileNetV2 model shows the most optimal balance between sensitivity and specificity across all classes. In **Figure 8**, the ROC-AUC curve specific to the proposed model (VGG16 + MobileNetV2) confirms its superior classification ability, with near-perfect AUC scores (≥0.99) across all classes. This indicates excellent model confidence and reliable disease differentiation, reaffirming its suitability for real-world guava disease diagnostics.

**Figure 9:**
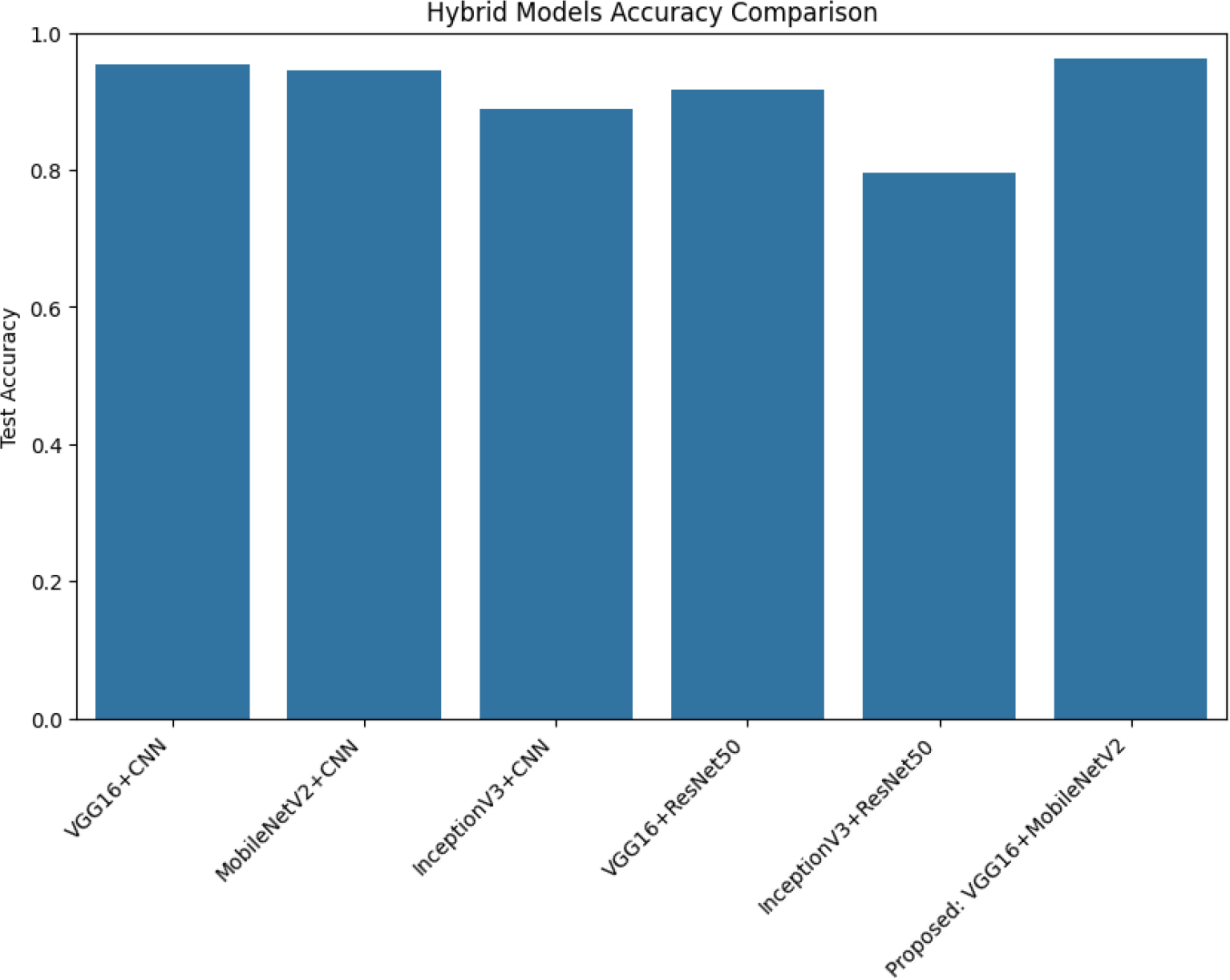
Test accuracy comparison of all hybrid models. The proposed VGG16 + MobileNetV2 hybrid achieves the highest accuracy among the six evaluated architectures.

**Figure 9** illustrates the comparative test accuracy of all hybrid models developed in this study. Among the six evaluated combinations, the proposed VGG16 + MobileNetV2 hybrid outperformed all others with the highest test accuracy, closely followed by VGG16 + CNN and MobileNetV2 + CNN. In contrast, the InceptionV3 + ResNet50 hybrid exhibited the lowest performance. This visual comparison underscores the superiority of combining VGG16’s deep texture extraction with MobileNetV2’s efficient spatial encoding.

**Figure 10:**
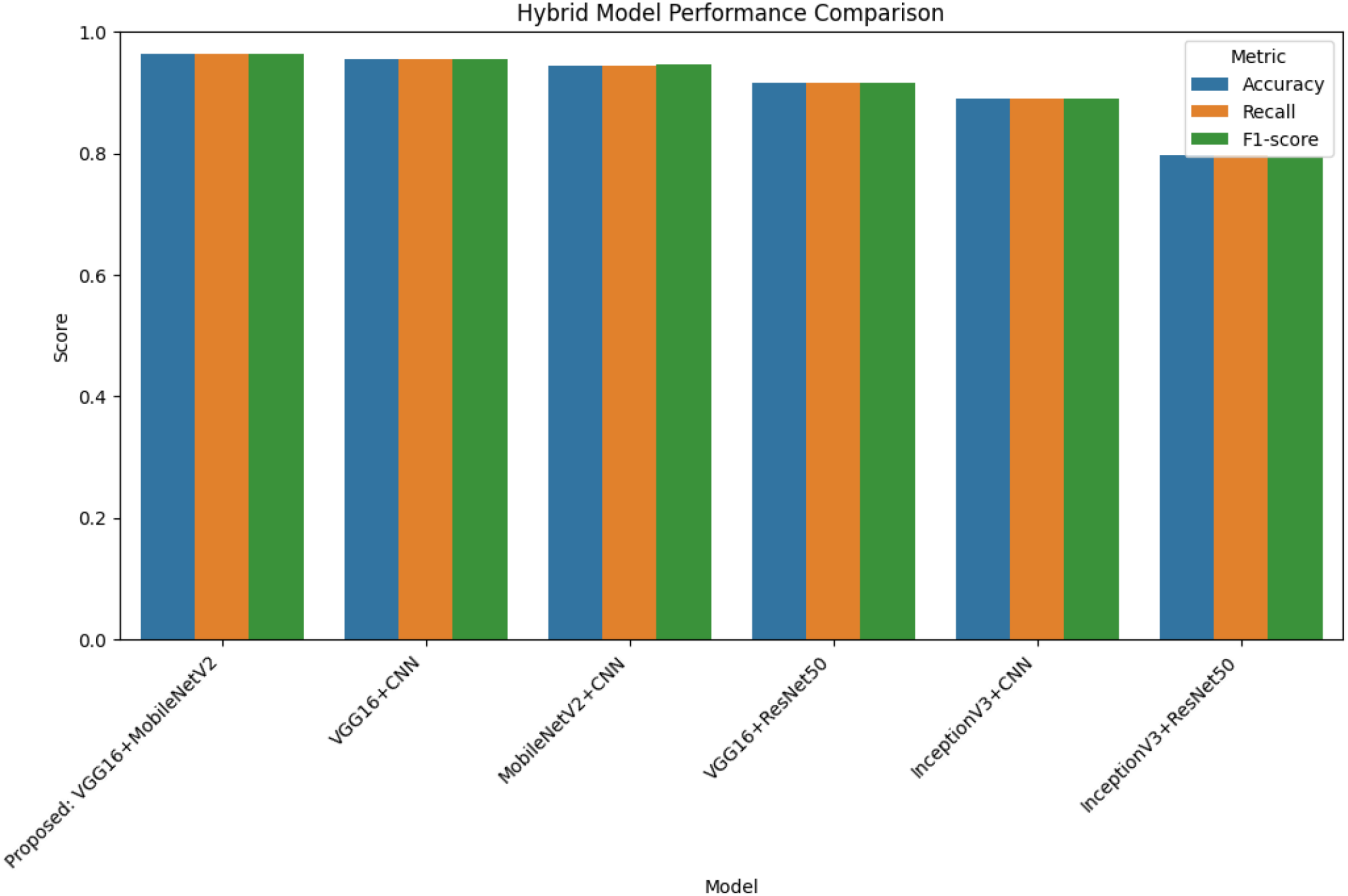
Performance comparison of all hybrid models based on accuracy, recall, and F1-score, illustrating the superiority of the proposed VGG16 + MobileNetV2 model.

The overall performance comparison outlined in **Figure 10** indicates the accuracy of each, and every hybrid model in terms of three important metrics namely accuracy, recall, and F1-score. Proposed VGG16 + MobileNetV2 hybrid has the highest score among all the parameters of the evaluation, and the proposed model is more accurate than others. Though the performance of VGG16 + CNN and MobileNetV2 + CNN patterns shows the same level of performance, which is high and balanced, InceptionV3 + ResNet50 pattern has an overall lower score, which creates a problem in extracting the complex category patterns of the diseases. Such a multi-metric testing backs the solidity and transferability of the suggested hybrid architecture.

**Figure 11:**
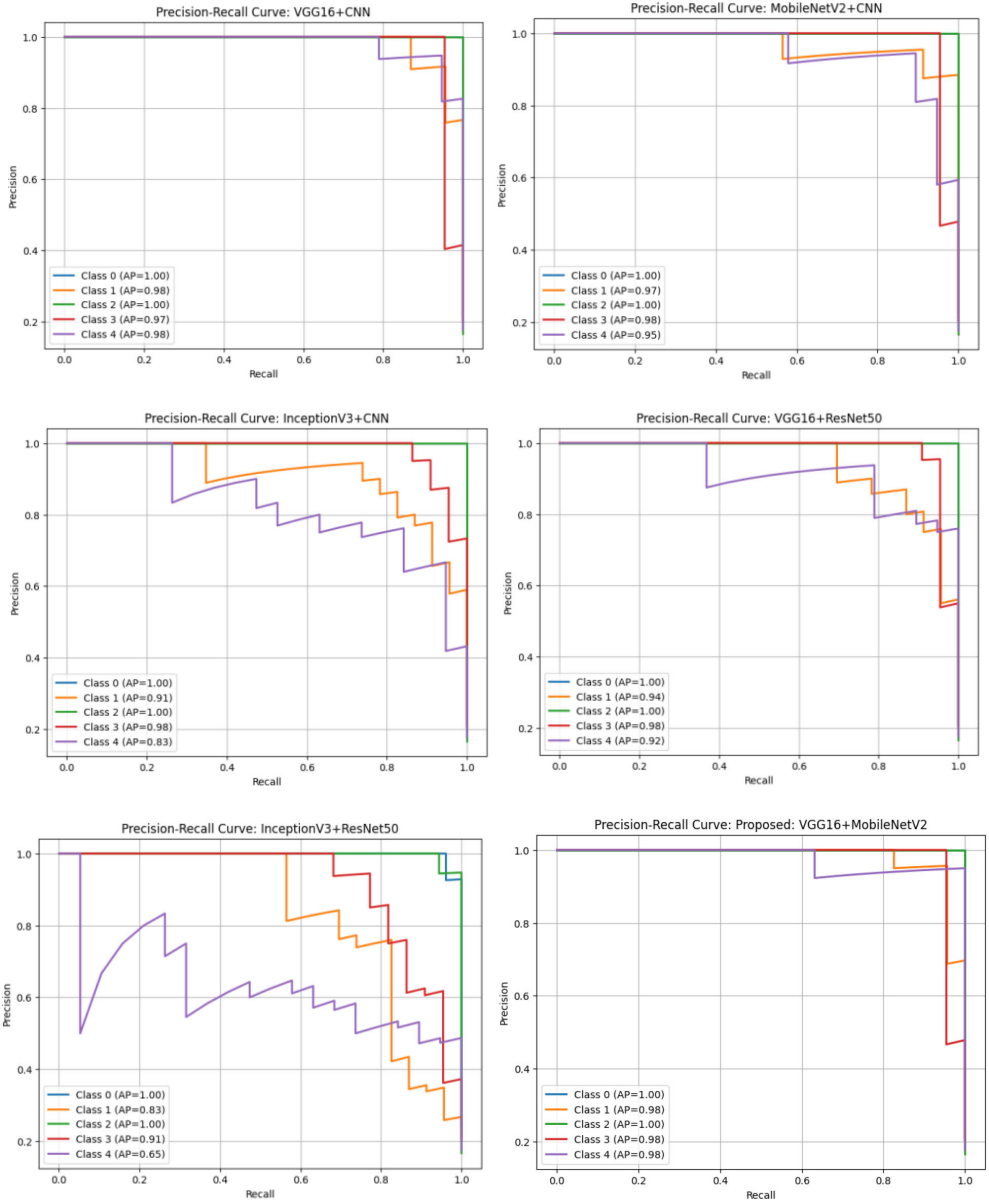
Multi-class Precision-Recall (PR) curve analysis across all hybrid models.

**Figure 11** gives a comparative picture of the multi-class Precision-Recall (PR) curves of all the six hybrid modules. Of these models, the proclaimed VGG16 + MobileNetV2 model has achieved a high level of average precision (AP) value in all five guava disease classes and is therefore getting optimum precision-recall balance. Contrastingly, InceptionV3 + ResNet50, and InceptionV3 + CNN exhibit significant reduction in precision on minority classes like Styler and Root Rot and Phytophthora for fewer reliable models with regard to class imbalance. The models VGG16 + CNN and MobileNetV2 + CNN are competitive, and yet they experience a slightly lower recall in some classes than the proposed model. All in all, real-world class distribution of the curves of PR once again confirms the increased sensitivity, generalization ability of the suggested hybrid model.

**Figure 12:**
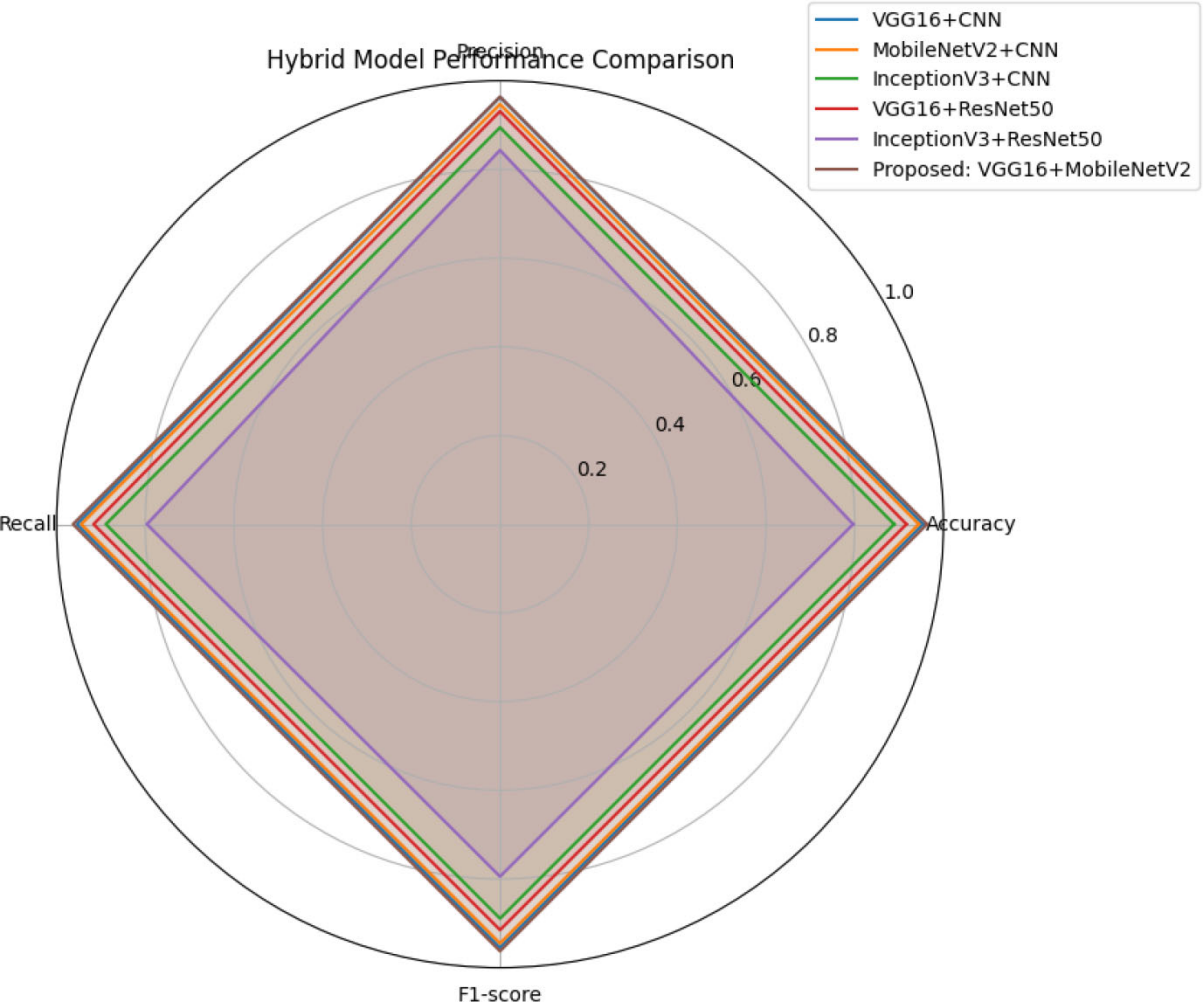
Radar chart showing comparative model performance across Accuracy, Precision, Recall, and F1-Score.

The radar chart in **Figure 12** provides a holistic comparison of the performance of multiple hybrid models across four core metrics: accuracy, precision, recall, and F1-score. The proposed VGG16 + MobileNetV2 hybrid model forms the most complete and balanced outer polygon, indicating consistently high performance across all evaluated dimensions. In contrast, models such as CNN + EfficientNetB0 and ResNet50 + EfficientNetB0 show significant contractions, particularly in recall and F1-score, reflecting suboptimal generalization. This visual reinforces the robustness of the proposed model in accurately and reliably classifying guava diseases across heterogeneous image samples.

**Figure 13:**
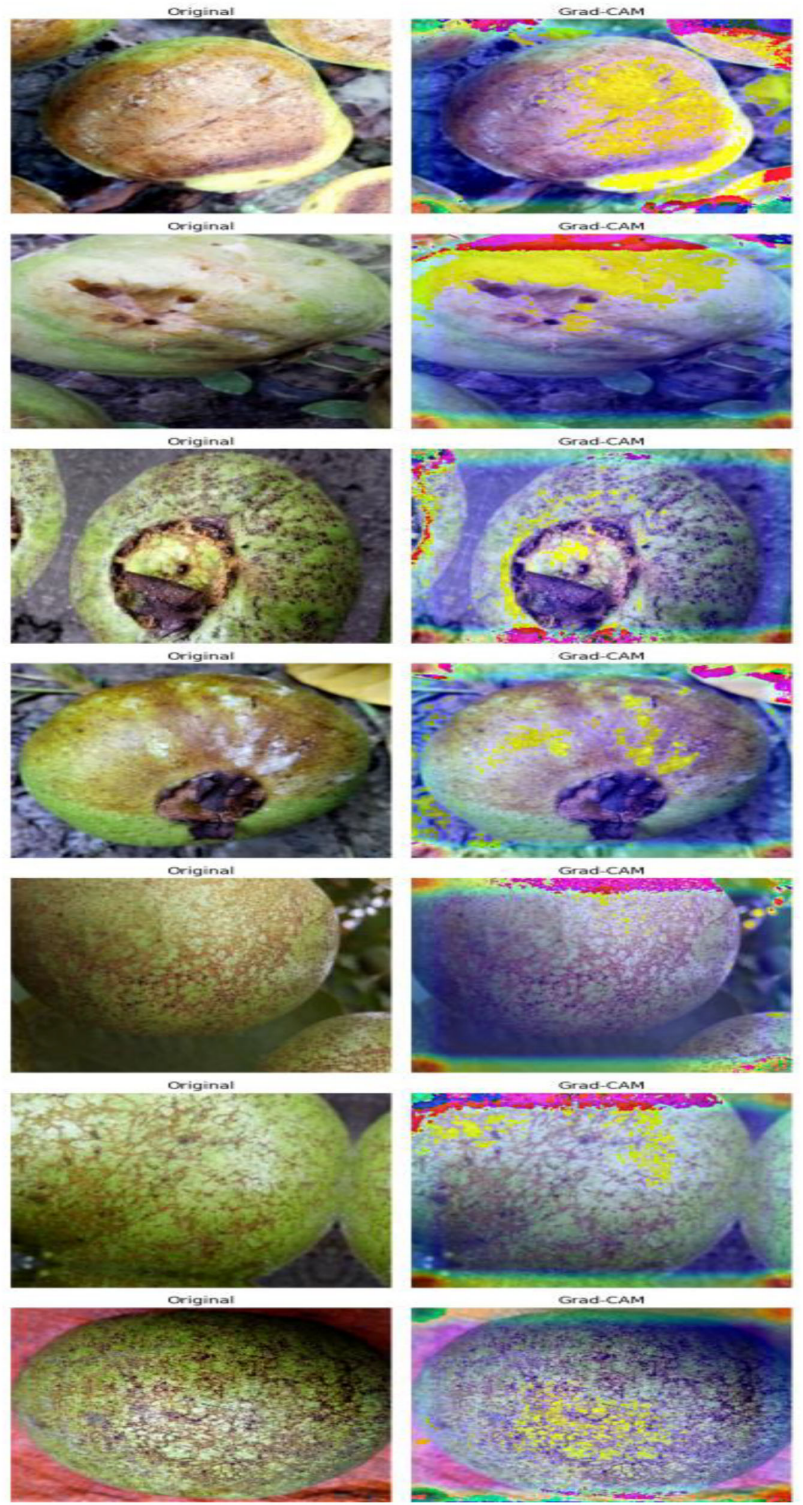
Grad-CAM visualizations for the proposed VGG16 + MobileNetV2 model showing original images alongside class activation maps, highlighting the critical disease regions influencing model predictions.

The Grad-CAM visualizations provided in **Figure 13** can give us much insight into how exactly the proposed hybrid model (VGG16 + MobileNetV2) makes decisions as the most important areas are highlighted that are related to the disease classification. The two images adjacent to one another compare the as is guava leaf or fruit with the corresponding Grad-CAM heatmap, the intensity of activation, which is varied in blue (thinner) to red/yellow (thicker), showing the zone most accountable to the model prediction. Remarkably, the model pays particular emphasis to symptomatic areas including necrotic lesions, fungal coloration, and texture deformations placing the tag to the fact that the learned characteristics correspond to those understandable by humans. Such visual interpretability does not only testify to the model reliability but also allows showing the situation to the field level in digital plant pathology, which agronomists will see to trust and use AI-based diagnostic tools.

The performance of the six fine-tuned hybrid deep learning models was rigorously evaluated across multiple metrics including accuracy, precision, recall, and F1-score. As shown in **Figure 3**, confusion matrices for each model illustrate their respective classification capabilities across the five guava disease categories. Among these, the proposed VGG16 + MobileNetV2 hybrid model (**Figure 4**) displayed the highest classification accuracy, correctly identifying all instances of ‘Disease Free’, ‘Red Rust’, and ‘Styler and Root’ with minimal misclassification for ‘Phytophthora’ and ‘Scab’. The heatmaps of class-wise metrics (**Figure 5**) provide a detailed comparison of the performance of all models, while the proposed model’s per-class metrics (**Figure 6**) further confirm its robustness, achieving near-perfect scores for all disease classes.

In terms of ROC-AUC evaluation, the collective model curves in **Figure 7** highlight varying performance across architectures, with several models experiencing minor drops in true positive rates at low false positive rates. In contrast, the proposed model’s ROC-AUC curve (**Figure 8**) maintained AUC values of 0.99–1.00 across all classes, demonstrating superior discriminatory power. Additionally, **Figures 9**, **10** visualize model performance comparisons via bar plots, affirming that the proposed hybrid outperforms others across all metrics. This is further substantiated by the radar chart in **Figure 12**, where the proposed model encompasses the widest area, representing consistently higher performance across all metrics. Finally, **Figure 13** demonstrates the effectiveness of Grad-CAM visualizations in providing model explainability, clearly highlighting the lesion areas most influential in classification decisions, thereby validating the reliability and transparency of the model in real-world diagnostic scenarios.

**Table 3** presents a comprehensive comparison of all fine-tuned hybrid models based on their classification performance on the guava disease dataset. As shown in **Table 3**, the proposed VGG16 + MobileNetV2 model outperformed all others, achieving the highest accuracy, precision, recall, and F1-score.

**Table 3:** Model performance comparison on guava fruit and leaf disease dataset.

Hybrid Model	Accuracy	Precision	Recall	F1-Score
VGG16 + CNN	0.95	0.96	0.95	0.95
MobileNetV2 + CNN	0.94	0.95	0.94	0.95
InceptionV3 + CNN	0.89	0.89	0.88	0.88
VGG16 + ResNet50	0.92	0.93	0.92	0.91
InceptionV3 + ResNet50	0.80	0.84	0.80	0.79
Proposed: VGG16 + MobileNetV2	0.96	0.96	0.96	0.96

### Novelty

Recent studies in guava disease classification using deep learning have focused on maximizing accuracy through large, augmented datasets or complex pre-trained models, often without incorporating interpretability. For example, Tewari et al. (2024) employed DenseNet169 on a 527-image dataset and achieved 99.62% accuracy, but without any explainable AI integration. Similarly, Mostafa et al. (2022) used a heavily augmented dataset of 2889 images to achieve 99.54% accuracy using ResNet-50. However, these models act as black boxes, limiting practical trust in predictions. Doutoum et al. (2023) used Efficient Net with 1834 images, reaching 94.93% accuracy again without interpretability.

In contrast, the current study introduces a novel, interpretable hybrid model (VGG16 + MobileNetV2) trained on the same real-world 527-image dataset as Tewari et al., but with an emphasis on both accuracy (96%) and explainability (via Grad-CAM) **Table 4**. By combining VGG16’s deep texture representation with MobileNetV2’s lightweight spatial encoding, the model achieves a strong balance of performance and practicality. Most importantly, this study integrates Grad-CAM visualizations, allowing human experts to interpret and validate the disease predictions. This makes the model more suitable for real-world agricultural use, especially where transparency and trust are essential. Hence, this work fills the crucial research gap between accuracy and interpretability in guava disease classification using small, realistic datasets.

**Table 4:** Comparative summary of recent deep learning studies on guava disease classification.

Study (Authors, Year)	Dataset (Size, Classes)	Model architecture	Performance metrics	XAI used?
Mostafa et al. (2022)	321 images (augmented to 2889), 5 classes	CNNs (AlexNet, GoogLeNet, ResNet-50, etc.)	ResNet-50: Accuracy 99.54%	No
Doutoum et al. (2023)	1834 guava leaf images, 5 classes	Transfer learning (EfficientNet best)	Accuracy 94.93%	No
Nandi et al. (2022)	~500+ images, guava leaves & fruits, 5 classes	GoogleNet, EfficientNet, quantized CNNs	Accuracy: 97–99%	No
Tewari et al. (2024)	527 images, 5 classes (same Kaggle dataset as yours)	DenseNet169 (transfer learning)	Accuracy: 99.62%	No
Proposed (This Study, 2024)	527 images, 5 classes (real fruit + leaf photos)	Hybrid VGG16 + MobileNetV2 + Grad-CAM	Accuracy: 96%; Precision: 96%; Recall: 96%; F1: 96%	Yes (Grad-CAM)

## Conclusion

In this study, we presented a robust and explainable deep learning framework to classify guava fruit and leaf diseases using hybrid CNN models, incorporating both traditional convolutional architectures and modern transfer learning strategies. Our research systematically compared six fine-tuned hybrid combinations, including VGG16, MobileNetV2, InceptionV3, ResNet50, and custom CNN backbones, trained and evaluated on a real-world guava disease dataset comprising 527 annotated images distributed across five disease classes: Disease Free, Phytophthora, Red Rust, Scab, and Styler and Root Rot.

Among the models evaluated, the proposed VGG16 + MobileNetV2 hybrid achieved superior performance, recording the highest classification accuracy of 96%, along with strong precision (0.96), recall (0.96), and F1-score (0.96), thereby validating the effectiveness of combining deep texture-based and lightweight spatial features. The integration of explainability via Grad-CAM provided valuable visual insights into the model’s decision-making process, enhancing transparency and supporting trust in AI-assisted disease diagnostics. Visualization tools such as confusion matrices, precision-recall heatmaps, ROC-AUC curves, and radar plots enabled a thorough comparative analysis and highlighted the diagnostic potential of our hybrid architecture.

This work contributes significantly to the domain of smart agriculture by offering an interpretable and scalable AI-driven solution for early guava disease detection. The proposed pipeline can be extended to other horticultural crops and real-time mobile diagnostic applications. Future research may explore the integration of hyperspectral or drone-captured imagery and the deployment of lightweight edge-optimized models for field-based implementation, further empowering farmers with real-time, automated plant health monitoring systems.

## Data Availability

The original contributions presented in the study are included in the article/supplementary material. Further inquiries can be directed to the corresponding author.
